# Safety and efficacy of osimertinib 160 mg daily given concurrently with a strong CYP3A4 inducer

**DOI:** 10.1016/j.rmcr.2025.102200

**Published:** 2025-04-01

**Authors:** Mostafa Aglan, Erin Spyropoulous, Joel Oster, Paul J. Hesketh, A.J. Piper-Vallillo

**Affiliations:** a Department of Internal Medicine, Lahey Hospital and Medical Center, 41 Mall Road, Burlington, MA, 01805, USA; b Lahey Hospital and Medical Center, 41 Mall Road, Burlington, MA, 01805, USA; c Department of Neurology, Tufts Medical Center, 260 Tremont Street, Boston, MA, 02116, USA; d Division of Hematology and Oncology, Lahey Hospital and Medical Center, 41 Mall Road, Burlington, MA, USA

**Keywords:** EGFR, Osimertinib, 160 mg, AED, Drug interaction

## Abstract

**Introduction:**

Osimertinib remains the standard first-line therapy for patients with advanced *EGFR*-mutant NSCLC, at least in part due to its improved CNS penetrance compared to earlier generation EGFR TKIs. Strong CYP3A4-inducing medications are known to reduce the effective concentration of osimertinib, prompting the recommendation to double the standard osimertinib dose from 80 to 160 mg daily. However, little is known about the real-world safety and efficacy of osimertinib given in combination with long term CYP3A4 inducer use. We detail, to our knowledge, the first reported case of a patient receiving an escalated osimertinib dosage concurrent with a potent CYP3A4 inducer.

**Case presentation:**

A 69-year-old-female with a long-standing history of a seizure disorder was diagnosed with stage IV EGFR exon 19 deletion positive lung adenocarcinoma. After a failed trial to wean the patient off phenytoin, osimertinib at a dose of 160 mg in combination with phenytoin was recommended based on existing clinical guidelines. She achieved a partial response and continues with stable disease for more than 32 months from initiation of osimertinib. Additionally, she tolerated osimertinib well with minimal side effects although with persistent dyspnea of unclear etiology.

**Conclusion:**

Our case illustrates that 160 mg of osimertinib administered concurrently with a strong CYP3A4 inducer can be given safely and with retained efficacy in treating CNS metastatic EGFR-positive non-small cell lung cancer.

## Highlights

• Patient with advanced EGFR Ex19del+ NSCLC required concurrent treated with osimertinib and a strong CYP3A4 inducer.
• Dose-escalated osimertinib (160 mg daily) co-administered with a CYP3A4 inducer provided durable disease control.
• Concurrent dosing was well-tolerated, with minimal side effects, supporting the feasibility of this dosing approach.

## 1 Introduction

Osimertinib, a third-generation, irreversible epidermal growth factor receptor tyrosine kinase inhibitor (EGFR TKI), remains the standard first-line systemic therapy for metastatic *EGFR*-mutant lung adenocarcinoma [1]. It exhibits activity against T790M, L858R, and exon 19 deletion mutations, and has demonstrated improved tolerability and survival compared to earlier generation EGFR TKIs. The improved efficacy of osimertinib over earlier generation EGFR TKIs is due, at least in part, to its improved CNS penetrance translating to better control of known CNS metastases and reducing the risk of CNS progression [2]. However, CNS penetrance appears to be dose-dependent such that drug interactions that decrease plasma concentrations of the active metabolite, AZ5104, are an important consideration, especially for patients with CNS disease at presentation [3,4]. When combined with a strong CYP3A4 inducer, one prospective study demonstrated that the AUC and Cmax of AZ5104, were reduced by 78 % and 82 %, respectively [5]. Based on this data, US label recommendations are to increase the osimertinib dose to 160 mg/day [6]. We present, to our knowledge, the first reported case of a patient treated with dose-escalated osimertinib while concurrently treated with an anti-epileptic drug (AED) known to be a strong inducer of CYP3A4.

## 2 Case presentation

**Fig. 1:**
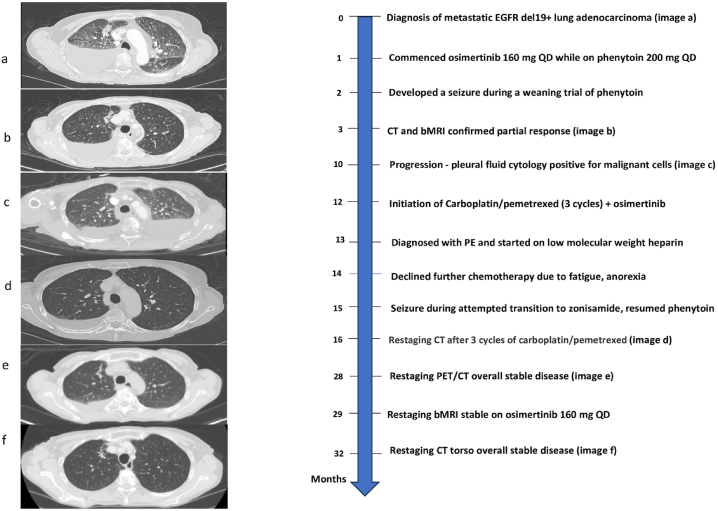
Legend – Timeline of events in months since diagnosis.

**Fig. 2:**
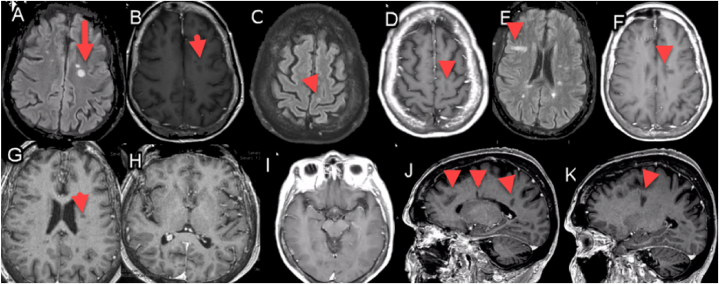
Legend –Representative neuroimages from most recent brain MRI demonstrating stable findings for an approximately 3-year period. Representative neuroimages from most recent brain MRI demonstrating stable findings for an approximately 3-year period. A: Axial flair showing prominent lesions. B: Axial T1 weighted neuroimaging with gadolinium showing no enhancement C: Axial flair with gadolinium showing a small cortical lesion along with D: T1 weighted imaging in a similar section as C. E: Additional axial flair MRI imaging at the level of the lateral ventricles. F: T1 with gadolinium enhanced MRI showing no definite enhancement along with additional sections at G and H highlighting a region at the level of the basal ganglia. I: Highlights an axial T1 with gadolinium section highlighting the preservation of mesial structures (hippocampi and temporal lobes) and J and K: sagittal T1 MRI selections with gadolinium highlighting lesions in periventricular and deep white matter locations.

A 69-year-old-female was diagnosed with stage IV lung adenocarcinoma with osseous and CNS metastases after experiencing persistent, worsening chest wall pain. A chest CT scan revealed numerous pulmonary nodules in a miliary pattern, along with an irregular area of consolidation in the right upper lobe, a large right-sided pleural effusion, and enlargement of lymph nodes in the left perivascular and right hilar regions as shown in Fig. 1 (image a). Subsequently, she underwent a right thoracentesis, with cytology confirming the presence of lung adenocarcinoma. Rapid EGFR PCR testing identified an activating exon 19 deletion. Further comprehensive staging scans, including brain MRI, identified brain metastases with two small enhancing areas in the right pre- and postcentral gyri, consistent with metastatic lesions as shown in Fig. 2. Additionally, there were small metastases in the anterior aspect of the left temporal bone. MRI of the thoracic spine revealed multiple metastases in the thoracic spine, some of which extended into the epidural space at T3, causing moderate canal stenosis and dorsal cord flattening. At T8, there was mild canal stenosis, and a compression fracture with retropulsion was observed at T12. A recommendation was made to begin osimertinib.

Due to a long-standing seizure disorder, she had been treated with phenytoin since childhood having undergone previous unsuccessful trials of alternative AEDs. Unfortunately, an attempt to taper the phenytoin while cross-titrating levetiracetam resulted in a tonic clonic seizure. Subsequently, she was maintained on 200 mg phenytoin daily (with higher dosing 1–2 times weekly periodically during her course) with plasma levels consistently in the therapeutic range (10–20 ucg/mL). Ethosuximide 500 mg twice daily and levetiracetam 1000 mg twice daily were continued concurrent with phenytoin. Given the ongoing need to maintain treatment with phenytoin, a recommendation was made to begin osimertinib at 160 mg daily (standard dose 80 mg daily) as treatment for her lung cancer. No seizures reoccurred while receiving phenytoin and osimertinib. All of her other medications were free of significant interactions with osimertinib (low molecular weight heparin 1.5 mg/kg daily and vaginal estradiol).

Her first restaging imaging performed after three months on osimertinib (CT chest abdomen pelvis with contrast and brain MRI) demonstrated a partial response, with a decrease in size of both the right upper lobe neoplasm and right hilar lymphadenopathy, in addition to stable osseous and CNS disease as shown in Fig. 1 (image b) and Fig. 2.

After nine months on osimertinib, 160 mg daily, she developed a recurrent right pleural effusion that required pleural catheter insertion with pleural fluid cytology repeatedly negative for malignant cells. She ultimately underwent right pleurodesis with resolution of the right effusion. However, 10 months after osimertinib initiation, her fourth thoracentesis performed on the left side was positive for malignant cells confirming the first definitive evidence of disease progression on osimertinib. A trial of carboplatin 4 AUC and pemetrexed 500 mg/m2 was recommended because of concern that the bilateral effusions were resulting in persistent and significant shortness of breath in combination with continuation of osimertinib 160 mg QD (given continued CNS disease control). An alternative approach—continuing osimertinib monotherapy—was considered but deemed suboptimal due to symptomatic disease progression and concern that progression elsewhere may ensue. Restaging studies, including CT torso and brain MRI, obtained after three cycles of carboplatin/pemetrexed demonstrated overall stable disease as shown in Fig. 1 (images c and d).

She developed a symptomatic pulmonary embolism following three cycles of chemotherapy and elected to discontinue chemotherapy due to worsening anorexia, fatigue, as well as the development of progressive cytopenias. In the absence of preceding trauma or DVT, the etiology of her pulmonary embolism was unclear, but the greatest risk factor was underlying lung cancer. She was started on weight-based low molecular weight heparin once daily with resolution of the pulmonary emboli confirmed on subsequent imaging.

She has since continued osimertinib 160 mg in combination with phenytoin. At 32 months after initiating osimertinib, a CT of the chest, abdomen, and pelvis with contrast demonstrated overall stable visceral, and osseous disease. Notably, the left pleural effusion has completely resolved, while the right pleural effusion remained stable in size as shown in Fig. 1 (image f). No new lesions or metastatic sites were detected. She tolerated osimertinib well, developing nail changes with increasing brittleness (grade 1), but experiencing no significant rash, mucositis, or diarrhea. Seizure activity has been well controlled and phenytoin levels have remained stable.

She has noted persistent and limiting dyspnea throughout her treatment course. The dyspnea did improve after initial treatment for the pulmonary embolism but subsequently returned with imaging demonstrating no recurrence of pulmonary emboli. Echocardiogram, exercise stress testing, and cardiopulmonary exercise test have been normal. Pulmonary function tests (PFTs) were performed, for the patient, who was 63 inches in height and weighs 106 lbs. The results showed an FEV1 of 1.92 L, FVC of 2.52 L, FEV1/FVC ratio of 0.76, normal spirometry, TLC, and a mild reduction in DLCO, altogether suggesting a diagnosis of COPD/emphysema according to her pulmonologist. Oxygen saturation remains normal at rest and with exertion. A one-week trial of osimertinib dose reduction to 80 mg daily while continuing phenytoin resulted in no significant improvement of dyspnea after which she resumed osimertinib 160 mg daily.

## 3 Discussion

This case report provides evidence of the safety and feasibility of co-administration of osimertinib with a strong CYP3A4 inducer through escalation of the osimertinib dose to 160 mg daily. Our patient achieved prolonged disease control on osimertinib treatment, with disease progression that later stabilized following three cycles carboplatin/pemetrexed with continued osimertinib at a dose of 160 mg daily. Notably, the higher osimertinib dosage did not result in any significant adverse effects. These findings suggest that adjusting the osimertinib dosage to 160 mg can be a viable strategy for maintaining treatment efficacy when concurrent use of a known potent CYP3A4 inducer is required.

The U.S. Food and Drug Administration label guidelines recommend avoiding concurrent use of strong CYP3A inducers with osimertinib whenever possible. In cases where strong CYP3A4 inducers cannot be avoided, an increased osimertinib dose of 160 mg/day is recommended during concurrent use, with a return to the standard dose (80 mg/day) three weeks after discontinuation of the inducer [6]. The recommended dose adjustment is based on a single, open-label, prospective drug-interaction study in which the pharmacokinetics (PK) of osimertinib in combination with a strong CYP3A4 inducer was analyzed [5]. In the study, 40 participants were treated with osimertinib (80 mg/day for 77 days) combined with the strong CYP3A4 inducer, rifampicin (600 mg/day for 21 days), resulting in a 78 % reduction in osimertinib AUC and an 82 % reduction in AUC and 78 % reduction in Cmax for AZ5104. Ethnicity did not appear to influence osimertinib exposure, despite a higher proportion of white patients in the rifampicin study.

To our knowledge, this represents the first published case of the real-world safety and efficacy of this strategy being utilized throughout a prolonged duration of treatment. One other published case of osimertinib given at 160 mg daily in combination with rifampicin (given for refractory wound infection) noted the safety of this combination, but the patient experienced primary progression after only 2 months of treatment [7].

## 4 Conclusion

Our case provides evidence that osimertinib 160 mg can be safely administered concurrently with a known CYP3A4 inducer while preserving efficacy in CNS metastatic EGFR positive non-small cell lung cancer.

## CRediT authorship contribution statement

**Mostafa Aglan:** Writing – original draft. **Erin Spyropoulous:** Writing – review & editing. **Joel Oster:** Writing – review & editing. **Paul J. Hesketh:** Writing – review & editing. **A.J. Piper-Vallillo:** Writing – original draft.

## Declaration of competing interest

The authors declare that they have no competing interests.
